# A Six-Year Gynecological Follow-Up of Immunosuppressed Women with a High-Risk Human Papillomavirus Infection

**DOI:** 10.3390/ijerph19063531

**Published:** 2022-03-16

**Authors:** Aleksandra Wielgos, Bronisława Pietrzak, Barbara Suchonska, Mariusz Sikora, Lidia Rudnicka, Miroslaw Wielgos

**Affiliations:** 1 Department of Dermatology, Medical University of Warsaw, 02-008 Warsaw, Poland; lidiarudnicka@gmail.com; 2 1st Department of Obstetrics and Gynecology, Medical University of Warsaw, 02-115 Warsaw, Poland; bpietrzak@wum.edu.pl (B.P.); barbara.suchonska@wp.pl (B.S.); miroslaw.wielgos@wum.edu.pl (M.W.); 3 National Institute of Geriatrics, Rheumatology and Rehabilitation, 02-006 Warsaw, Poland; drmariuszsikora@gmail.com

**Keywords:** HPV, human papillomavirus, immunosuppression, transplantation, cervical cancer, self-sampling

## Abstract

Immunocompromised women are at an increased risk of developing malignancies, especially those that are viral-induced, such as invasive cervical cancer caused by the human papillomavirus (HPV). The aim of the study was to describe gynecological follow-up of women undergoing chronic immunosuppressive therapy for various reasons (e.g., kidney/liver transplant, systemic lupus erythematosus), diagnosed with a high-risk HPV (hrHPV) infection based on a self-sampling test. Twenty-six hrHPV-positive women were invited to take part in a gynecological follow-up, including a visual assessment of the anogenital region, two-handed gynecological examination, and cervical cytology as well as a colposcopy and cervical biopsy when necessary. Four women declined taking part in the study. Over six years of observation, low-grade squamous intraepithelial lesions (LSIL) were detected at least once in 7/22 women (31.8%), and a cervical intraepithelial lesion 1 (CIN 1) histopathologic result was obtained five times in 3/22 women. No cases of high-grade squamous intraepithelial lesions, CIN 2/3, or invasive cervical cancers were observed. Loop electrosurgical excision procedure (LEEP) was performed in three patients. As immunocompromised women are prone to persistent hrHPV infections, they should be under strict gynecological supervision because only vigilant surveillance enables fast detection and treatment of early dysplasia and, therefore, provides a chance for the reduction of the cervical cancer burden.

## 1. Introduction

Immunocompromise, including that of iatrogenic etiology due to use of immunosuppressive drugs, e.g., in solid organ transplant recipients (SOTR) or patients with autoimmune diseases, is a well-established risk factor of anogenital malignancies’ development, especially those that are viral-induced. A link has been described between immunosuppressive medication dose, therapy duration, and development of malignancies [1]. The most prevalent of those malignancies among women is cervical cancer, associated with a high-risk human papillomavirus (hrHPV) infection [2]. Despite increasing interest in this field, opinions vary whether hrHPV infections are more frequent among immunocompromised women or the incidence is comparable to that in healthy individuals [3,4,5,6,7,8,9,10,11]. It is, however, accepted that HPV infections that are usually transient in immunocompetent individuals tend to be persistent among the immunocompromised ones, leading to faster oncogenesis [12]. Other factors that predispose to persistent hrHPV infections and dysplasia development include smoking, genetic factors, and sexually transmitted diseases; these may also increase the risk in immunocompromised women [13].

The most studied population of noniatrogenically immunocompromised women are human immune deficiency (HIV)-positive women in which a higher incidence of hrHPV and reduced hrHPV clearance is observed as well as an increased incidence of progression from normal cervical cytology to a low-grade squamous intraepithelial lesion (LSIL) and high-grade squamous intraepithelial lesion (HSIL) in hrHPV-positive women when compared to healthy controls [14].

With the improvement of immunosuppressive regimens and the reduction of rejection rates, the life expectancy of SOTR increased and, therefore, nowadays, malignancies seem to have a crucial impact on patients’ survival. Taking into consideration the higher risk of malignancy development, guidelines (e.g., European Best Practice Guidelines Expert Group on Renal Transplantation) suggest performing at least annual gynecological examinations of immunosuppressed women in order to perform regular cervical cancer screening [15]. As HPV DNA testing presents higher sensitivity when compared to cervical cytology, its use in cervical cancer (CC) screening is increasing. To improve patients’ acceptance and accessibility of the screening as well as participation rates among screening nonresponders, HPV DNA self-testing methods have been invented, and their accuracy has been approved as comparable with clinician-obtained samples [16].

The aim of the presented study was to describe the results of a gynecological follow-up, including cervical cytology, colposcopy, and histopathologic examinations of immunosuppressed women with a history of hrHPV infection diagnosis, based on a self-sampling test, with a special emphasis on the incidence of genital dysplasia.

## 2. Materials and Methods

In the primary phase of the study, 90 immunocompromised women hospitalized in the Institute of Transplantology, Medical University of Warsaw, Poland and consulted in the outpatient clinics of the First Department of Obstetrics and Gynecology and Department of General, Transplant and Liver Surgery, Medical University of Warsaw, were asked to self-collect a cervicovaginal specimen, using the Evalyn Brush^®^ device (Rovers Medical Devices B.V., Oss, The Netherlands) [17]. The samples were later examined in search for HPV DNA with a Q|Amp virus kit (Qiagen, Hilden, Germany) based on polymerase chain reaction (PCR). Afterwards, genotyping was performed using the Genotyping kit HPV GP version 2 (Labo Bio-medical Products B.V., Rijswijk ZH, The Netherlands) and allowing the qualitative identification of HPV type 6, 11, 16, 18, 26, 30, 31, 33, 35, 39, 45, 51, 52, 53, 56, 58, 59, 66, 67, 68, 70, 73, and 82. Genotypes that were impossible to identify with the Genotyping kit were considered as type “X”. Genotypes 6, 11, 30, and “X” were considered low risk, and genotypes 16, 18, 31, 33, 35, 39, 45, 51, 52, 56, 58, and 59 were considered high risk (hrHPV); genotypes 26, 53, 66, 67, 68, 70, 73, and 82 were considered possible/probable high risk and classified as hrHPV for analytical purposes [10,18]. All patients enrolled met the primary inclusion criteria of chronic immunosuppression (>3 months) for various reasons (e.g., kidney/liver transplant, systemic lupus erythematosus) and were aged 18–70 years. The exclusion criteria constituted of menstruation on the day of a possible enrollment or a history of cervical cancer. The patients invited to participate in the study were chosen randomly based on their presence in the Institute of Transplantology or the outpatient clinics during the enrollment period as there is no nationwide registry of immunocompromised patients in Poland [19].

Twenty-six of the above-mentioned women tested positive for an hrHPV infection (28.9% of the primary study group) and were invited to take part in a gynecological follow-up in the outpatient clinic of the First Department of Obstetrics and Gynecology, Medical University of Warsaw. 4/26 women declined participation in the follow-up at baseline.

At a follow-up gynecological visit, a visual inspection of the anogenital region as well as bimanual gynecological examination (in order to check the size and location of patient’s uterus and ovaries) was performed. All the patients had a cervical (or vaginal vault in case of a history of hysterectomy) cytology test performed at baseline. Colposcopy was offered at baseline to all the patients, except for one patient who was a virgin, and was performed in all the patients with an abnormal cervical cytology result and all the other patients who agreed to have it performed despite a normal cytology result. A cervical colposcopy-guided biopsy was performed in all the patients with an abnormal colposcopy, and an endocervical biopsy was obtained in the patients with abnormalities in the colposcopy image. All the patients were advised to schedule gynecological visits annually and to repeat gynecological examination and cervical cytology, except for the patients with abnormal results of cervical cytology test or histopathology, who were told to have their appointments every six months. Performing colposcopy with or without a cervical biopsy in the follow-up period was restricted to patients with an abnormal cervical cytology result in the particular year of observation. Immunocompromised patients with LSIL/cervical intraepithelial neoplasia 1 (CIN 1) obtained repeatedly (at least twice) were referred for excisional procedures (loop electrosurgical excision procedure (LEEP)/large loop excision of the transformation zone (LLETZ)) as a group of high risk of cervical malignancy. This approach was implemented as a diagnostic method and to avoid further development of neoplasia in immunosuppressed patients. The follow-up lasted six years, but the patients still remain under the supervision of the gynecological outpatient clinic after the closing period of the study. The groups’ characteristics are presented in Table 1.

The data from the follow-up period, including cervical cytology, colposcopy, and histopathologic results, were collected, and a descriptive analysis of the obtained material was performed.

The study design was approved by the Bioethical Committee at the Medical University of Warsaw, Poland (KB/102/2015; 5 May 2015). Women enrolled in the study received oral and written information about the details and purpose of the study and provided written consent for participation.

## 3. Results

**Scheme 1:**
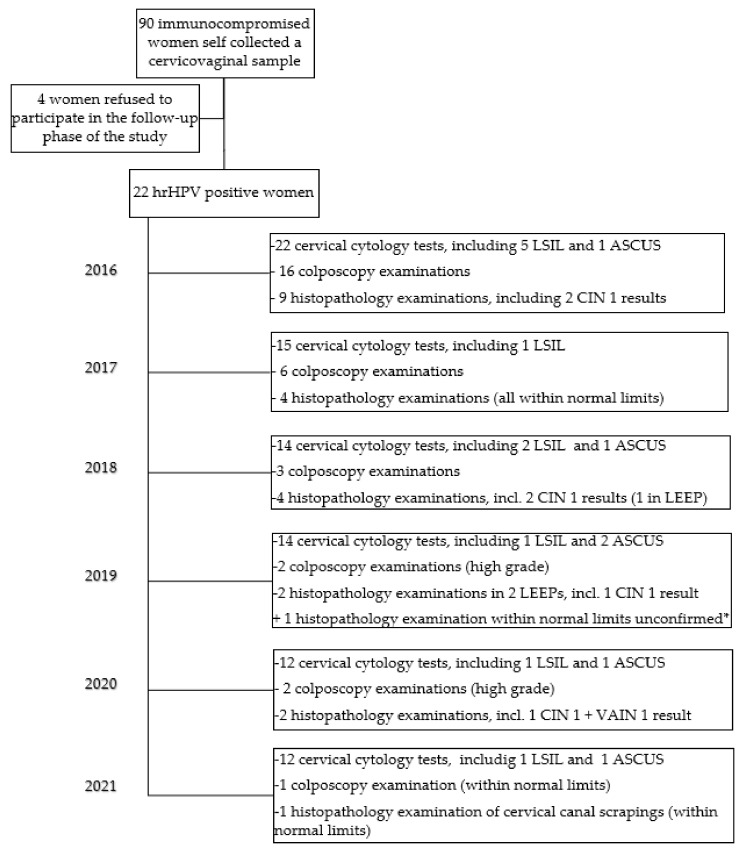
Results of the six-year follow-up gynecological examinations. hrHPV: high-risk human papillomavirus; LSIL: low-grade squamous intraepithelial lesion; ASCUS: atypical squamous cells of undetermined significance; CIN 1: cervical squamous intraepithelial neoplasia 1; VAIN 1: vaginal intraepithelial neoplasia 1; LEEP: loop electrosurgical excision procedure. * The result of a biopsy obtained in a different department, as reported by the patient.

The detailed information on the results of the follow-up is summarized in Scheme 1.

The majority of patients did not participate in the follow-up visits annually as advised. During the study period, only two (9%) patients appeared for the gynecological examination at least annually and have not omitted a cervical cytology in any year. Eight patients (36.4%) participated in selected five years of the follow-up, four patients (18.2%) in four years, six patients (27.3%) in three years, one (4.5%) in two years, and one (4.5%) in one year only. However, the patient who participated in two years of the follow-up (No 23) died during the observation period, but the cause of death was not related to gynecological status.

The detailed information on the abnormal cervical cytology test results, LEEP performed, and histopathological results of the six-year CC screening is presented in Table 2.

Over the six years of observation, LSIL was detected at least once in seven out of 22 women (31.8%); patient No 5 had LSIL in four years of observation and No 17 in two years. Patients No 13 and No 17, with a CIN 1 result in the cervical biopsy in 2016, were primarily qualified for LEEP but disqualified after a satisfying colposcopy control. The patient No 5, who already had a LEEP procedure in 2019, was diagnosed with CIN 1 altogether with vaginal intraepithelial neoplasia 1 (VAIN 1) in 2020, but the patient has not appeared for a follow-up in 2021, similar to patient No 17 with a history of LEEP in 2018 in whom an ASCUS result was present in early 2021.

There were no HSIL lesions in the examined group. None of the patients was diagnosed with cervical squamous intraepithelial neoplasia grade 2 or 3 (CIN 2/3) nor invasive cervical cancer during the follow-up.

Condylomata acuminata were present in the anogenital region of two of the hrHPV-positive women (No 5 and No 26).

## 4. Discussion

A considerable portion of cumulative LSIL results (31.8%; 7/22) was detected during the six years of follow-up after the diagnosis of hrHPV. This might be explained by the fact that our cohort consisted only of selected hrHPV-positive immunocompromised females as our result is consistent with the 33.3% of LSIL detected in five out of 10 hrHPV-positive women with abnormal cervical cytology from a cohort of American renal transplant recipients (RTR) from Mayo Clinic, described by Long et al. These data are, however, difficult to compare as Long et al. presented only the data for hrHPV patients at one year post transplantation [20].

The proportion of LSIL results in the whole registry-based cohort of Long et al. was reported to be 16.6% (76/459), which is consistent with 13.9% (23/165) of LSIL in the pre-study cytology (vs. 3.6% in a cytology taken during the study) in Brazilian RTR, described by Klitzke et al., and 14.5% (9/62) of LSIL in Mexican RTR, described by Parra–Avila et al. [20,21,22]. On the other hand, Roensbo et al. reported LSIL in 4/60 (6.7%) and HSIL in 3/60 (5.0%) Danish RTR and bone marrow transplant recipients [11]. Parra–Avila et al. reported HSIL in 2/62 (3.2%) RTR [22]. The high prevalence of precancerous cervical lesions, e.g., LSIL, is explained by the authors by the fact that spontaneous regression of such lesions is less probable in immunocompromised females than in healthy individuals [12].

Despite a significant proportion of detected LSIL cases, no cases of CIN 2/3 or cancer were discovered during the study period. Such predisposition for LSIL and CIN 1 was also observed by Klitzke et al., and the authors emphasized that this did not influence the morbidity and mortality in this group of patients [21]. This might be a result of a smart detection and treatment of premalignancies (e.g., performing LEEP), as suggested by Engels et al. [23].

In a study conducted in the Netherlands by Meeuwis et al., CIN histology was reported in 3.6% (8/224) of the RTR, which reflects a two- to sixfold risk when compared to controls [24]. The prevalence of genital dysplasia in the gynecological follow-up of immunocompromised females (in this case RTR) was also described by Marshalek et al., who reported 5.7% of CIN among Austrian RTR, with 3.4% being CIN 1. However, the status of hrHPV infections among the patients from that cohort and the number of abnormal cytology results remain unknown [13]. In a population-based study on female RTR in Denmark over the 20 years of follow-up, Reinholdt et al. reported CIN 2/3 in 59/14,017 RTR when compared to 1542/910,648 controls (HR 2.1) and CC in 9/15,055 cases when compared to 211/964,349 controls (HR 2.8) [25]. Long et al. described CIN 2 in 17 (3.7%) and CIN 3 in 9 (1.96%) out of 459 RTR [20]. In our cohort, no CIN 2/3 were detected.

None of the patients with CIN detected during the follow-up period in our cohort was positive for types 16 and 18, which are considered to be the highest potential for carcinogenesis; this, however, might be due to a small sample size [26,27].

Contrasting data are presented by authors in terms of the time interval after transplantation and the highest risk for cervical precancerous lesions development. Klitzke et al. observed the highest prevalence three to four years after transplantation, Marshalek et al. noted four to five years, Origoni et al. noted three to six years, and Meeuwis et al. noted 12.3 years after transplantation [4,13,21,24].

A very low number of females from the study group attended gynecological appointments on a regular basis, at least once a year at 9.1%, when compared to 82.7% described by Marshalek et al. The authors, however, pointed out that a very good compliance level for the CC screening program is also observed in the general Austrian population, which reflects the attendance of the study participants [13]. In contrast, the implementation of a preventive CC screening program financed by the Polish National Health Fund was reported as 12.6% on 1st January 2022 (the number does not include cervical cytology tests performed in private offices) [28].

The nonadherence of the study participants to the traditional clinician-obtained cervical cytology-based screening model established as a follow-up method may support the idea of HPV self-testing as a possibility for increasing the screening uptake, especially under the circumstances of the global COVID-19 pandemic. Therefore, HPV self-sampling should be considered a promising method of follow-up in such groups of nonresponders [17]. Meta-analyses show that HPV self-testing is a method of sensitivity comparable to clinician-obtained samples that is well accepted by women and enables the achievement of higher response rates when compared with traditional invitation-based testing [29,30]. Therefore, self-sampling has recently been included in the World Health Organization guideline for screening and treatment of cervical precancer lesions for CC prevention as well as in the guidelines for CC screening in the current pandemic by the Polish Society of Gynecologists and Obstetricians and the Polish Society of Colposcopy and Cervical Pathophysiology [31,32]. HPV self-testing was also mentioned as a CC screening possibility in the guideline update from the American Cancer Society; however, it was not recommended as it had not yet been approved by the Food and Drugs Administration [33].

It is worth emphasizing that this is a first prospective study describing gynecologic observation of hrHPV-positive, immunocompromised Polish women and one of the few studies available so far giving an insight on a relatively long follow-up period in a group of iatrogenically immunosupressed hrHPV-positive women in general. 

The limitation of the study is definitively a small study group, lack of hrHPV status verification during the follow-up, heterogenicity of reasons for immunosuppression, and immunosuppressive regimens. The cohort consisted, however, of selected individuals with both chronic immunosuppression and hrHPV. Unfortunately, there is no nationwide registry in Poland available enabling easy access to transplantation data.

Further studies on a bigger cohort of immunocompromised Polish women would, however, be necessary to draw conclusions that could support determining appropriate CC screening intervals in this population.

## 5. Conclusions

As immunocompromised females are at a higher risk of a persistent hrHPV infection, they should be carefully monitored in search for cervical dysplasia. Only a vigilant screening enables healthcare providers to detect cervical malignancy at the earliest stage possible and, therefore, reduce CC mortality. Therefore, immunocompromised patients should be monitored at least annually, and an individual follow-up schedule should be created for those individuals in whom abnormal examination results are obtained to best address the particular needs of each patient. When possible, the follow-up in this group should be based on both cervical cytology and HPV testing, which would enable monitoring of the hrHPV infections present at baseline but also detection of new infections. HPV self-sampling is an important alternative for clinician-obtained samples, especially in the situation of a pandemic, which is included in the WHO guidelines. Further studies are necessary to establish a screening approach that would be both effective and convenient to patients. Emphasis should be placed on education in this group of patients to increase the awareness of the higher risk of malignancy and the importance of regular gynecologic surveillance in order to improve the CC screening uptake.

## Figures and Tables

**Table 1:** Study group’s characteristics.

Patient No	Age at Enrollment (Years)	Reason for Immunosuppressive Therapy/ Primary Disease	Immuno-Therapy Duration at Enrollment (Months)	Immunosuppressive Therapy	Detected HPV Types
1	65	LT/HCV infection	132	MMF, CsA, steroids	66
2	60	RT/chronic glomerulonephritis	14	MMF, TAC, steroids	16
3	35	RT	132	TAC, steroids	45
4	35	LT/Budd-Chiari syndrome	23	TAC, steroids	45
5 **	38	RT/pancreas transplant	156	MMF, TAC, steroids	56, 66
6 *	32	RT	132	unknown	31, 45
7	43	LT/HCV infection	120	MMF, CsA, steroids	16, 31
8 *	35	SLE	288	MMF, AZA, steroids	16,31
9 *	32	Chronic glomerulonephritis	84	CsA, steroids	67
10	67	RT/polycystic kidney	48	TAC, steroids	33
11 *	68	RT/chronic glomerulonephritis	156	MMF, steroids	16
12	64	LT/ HCV infection	7	MMF, CsA, steroids	16, 18, 45, 67
13	41	RT/medullary sponge kidney	144	MMF, CsA	16, 18, 31, 45, 67
14	42	RT/chronic glomerulonephritis	84	MMF, TAC, steroids	16, 34
15	38	RT/polycystic kidney	118	MMF, steroids, everolimus	31
16	38	RT/granulomatosis with polyangiitis (GPA)	159	Mycophenolate sodium, TAC, steroids	33
17 **	37	RT/chronic glomerulonephritis	3	MMF, TAC, steroids	33
18	30	LT/AIH	90	MMF, TAC, steroids	31
19	46	RT	129	MMF, TAC, steroids	16, 67
21	59	RT	46	MMF, TAC, steroids	67
21	64	RT/polycystic kidney	180	TAC, steroids	45
22 **	52	RT/ DIC	96	MMF, TAC, steroids	31
23	39	RT/chronic glomerulonephritis	168	MMF, TAC, steroids	16
24	35	RT/ SLE	6	MMF, TAC, steroids	16, 56
25	58	RT	136	MMF, CsA, steroids	16
26	20	LT/AIH	10	MMF, TAC, steroids	6, 66

RT: renal transplantation; LT: liver transplantation; SLE: systemic lupus erythematosus; HCV: hepatitis C virus; AIH: autoimmune hepatitis; DIC: disseminated intravascular coagulation; MMF: mycophenolate mofetil; CsA: cyclosporine A; TAC: tacrolimus; AZA: azathioprine; Steroids: prednisone or methylprednisolone. * Patients who declined to undergo gynecological follow up; ** Patients in whom LEEP was carried out.

**Table 2:** Abnormal cervical cytology and histopathological results during the follow-up.

Year	LSIL	LEEP	CIN 1
2016	5/22 (22.7%)	---	patient No 13 (in CB) with a satisfactory cervical cytology and low-grade colposcopy result patient No 17 (in CB) with a LSIL result and low-grade colposcopy result
2017	1/15 (6.7%)	---	
2018	2/14 (14.3%)	patient No 17 with LSIL and low-grade colposcopy findings	patient No 17 (in LEEP)
2019	1/14 (7.1%)	patient No 5 with LSIL and high-grade colposcopy findings patient No 22 with ASCUS and high-grade colposcopy findings	patient No 5 (in LEEP)
2020	1/12 (8.3%)	---	patient No 5 (in CB) altogether with vaginal intraepithelial neoplasia 1(VAIN 1)
2021	1/12 (8.3%)	---	---

LSIL: low-grade squamous intraepithelial lesion; LEEP: loop electrosurgical excision procedure; CB: cervical biopsy; CIN 1: cervical squamous intraepithelial neoplasia 1; VAIN 1: vaginal intraepithelial neoplasia 1.

## Data Availability

The data presented in this study are available on request from the corresponding author. The data are not publicly available due to privacy reasons.
